# Preclinical assessment of pharmacokinetics and anticonvulsant activity of CBDTech, a novel orally administered cannabidiol (CBD) formulation for seizure and epilepsy

**DOI:** 10.1186/s42238-025-00322-7

**Published:** 2025-09-30

**Authors:** Jacob D. McDonald, Feng Zhou, Justyna Kulpa, Philip J. Kuehl

**Affiliations:** 1 Lovelace Biomedical, Albuquerque, NM USA; 2 Pharmaron LLC, Exton, PA USA; 3 Independent Scientist, Toronto, ON Canada

**Keywords:** Cannabidiol, CBD, PK, Absorption, Bioavailability, Brain, Excretion, Epilepsy, Seizure, MES

## Abstract

Oral cannabidiol (CBD) product use is increasing despite suboptimal pharmacokinetics (PK) of currently available formulations. This study aimed to investigate the PK of CBD formulated using the drug delivery technology DehydraTECH™, which is hypothesized to increase absorption by bypassing first-pass liver metabolism due to enhanced lipophilic composition. Anticonvulsant activity of the leading formulation was investigated in the maximal electroshock seizure (MES) model. For the PK studies, Sprague Dawley rats were orally administered 25 mg/kg CBD in MCT oil or test formulations incorporating DehydraTECH™ (*n* = 10 per group). Plasma, brain tissue and urine and feces samples were collected to determine comparative absorption, distribution, and excretion by liquid chromatography with tandem mass spectrometry (LC–MS/MS). For the efficacy studies, a series of experiments was conducted using the lead formulation (CBDtech) from the PK trial. Effective dose (ED) of CBDtech in comparison to Epidiolex® (50–100 mg/kg), time of peak efficacy (TPE), and median ED (ED50) were assessed in the acute MES model. Clinical observations, presence/absence of hind limb extension (HLE), and maximum seizure severity (MSS) were recorded. No abnormal clinical signs were observed following dosing in any study. Area under the curve from dosing to the last measurable concentration (AUC_last_) was 391 to 2708% improved following treatment with DehydraTECH™ formulations as compared with the MCT control (all *p* < 0.01). CBD was detected in brain, urine, and feces samples following all DehydraTECH™ treatments. Treatment with the ED of CBDtech (75 mg/kg) resulted in full protection (absence of HLE) in 66.6% of test subjects following MES test compared to 50% in the Epidiolex® group. The one-hour timepoint was determined to be the TPE for CBDtech; HLE was absent in 75% of animals and partial in 12.5% of animals. In comparison, in the Epidiolex® group HLE was absent in 50% of animals and partial in 12.5% of animals. The calculated ED50 was 75 mg/kg. Formulation of CBD with DehydraTECH™ resulted in improved bioavailability and efficacy in an acute seizure model. These findings contribute to a deeper understanding of CBD PK and will aid in the design of more effective CBD-based therapeutic interventions.

## Introduction

Cannabidiol (CBD) is a phytocannabinoid of increasing interest for its purported benefits in a variety of clinical conditions, including anti-depressant, anxiolytic, anti-inflammatory, and anticonvulsant effects (Atalay et al. 2019; Devinsky et al. 2014; García-Gutiérrez et al. 2020; Wright et al. 2020). Moreover, CBD has a favorable safety and tolerability profile, lacking the psychotropic effects more often associated with tetrahydrocannabinol (THC) (Grotenhermen et al. 2017). CBD is available in both over-the-counter supplements and as an FDA-approved drug treatment, Epidiolex®, for the treatment of seizures associated with rare childhood epilepsy syndromes (i.e., Lennox-Gastaut, Dravet, Tuberous Sclerosis Complex) (Rubin 2018).

While the oral administration of CBD-containing extracts and isolates is a common administration strategy, ingestion results in delayed absorption, low bioavailability (~ 6% of delivered dose), and highly variable pharmacokinetics (PK) due to CBD’s poor solubility in hydrophilic solvents and significant first-pass metabolism in the liver (Millar et al. 2018). CBD and its metabolites predominately undergo hepatobiliary elimination with ~ 82% of the total dose recovered in feces after a single administration (*Epidiolex Package Insert*, 2018). Considerable efforts have been made to improve the PK of orally-delivered cannabinoids to allow higher concentrations of CBD in plasma, including delivery via dietary fats, pharmaceutical lipid-based formulations, gelatin matrix pellets, and self-emulsifying drug delivery systems (SEDDS) (Atsmon et al. 2018; Knaub et al. 2019 Kok et al. 2022; Zgair et al. 2016). Importantly, increased bioavailability and more rapid absorption of CBD has been previously shown using an oral TurboCBD™ formulation as compared with an equivalent generic 90 mg capsule (i.e., recreational-equivalent dose) in young volunteers (Patrician et al. 2019). Advancing on this work, the patented DehydraTECH™ 2.0 CBD capsule has been developed by Lexaria Bioscience Corp. (Kelowna, BC, Canada). This patented technology is thought to increase CBD absorption by bypassing, in part or in full, first-pass liver metabolism due to its enhanced lipophilic composition (Kumric et al. 2022, 2023; Patrician et al. 2019).

This study examined the circulating concentration, brain distribution, and excretion of various orally administered CBD formulations utilizing DehydraTECH™ technology in rats. Moreover, the therapeutic efficacy of the leading formulation was determined in the maximal electroshock seizure (MES) model of epilepsy and compared to that of Epidiolex®. Data from the present study will provide a better understanding of how this lipid technology affects CBD absorption and function, and will aid in the design of more effective CBD-based therapeutic interventions for human clinical populations.

## Materials and methods

### Ethics statement

Animal work was conducted in the animal research facilities of Pharmaron (Exton, PA) for PK studies or Lovelace Biomedical (Albuquerque, NM) for efficacy studies, which were fully accredited by the Association for Assessment and Accreditation of Laboratory Animal Care (AAALAC). The studies complied with all applicable sections of the Final Rules of the Animal Welfare Act regulations (9 CFR Parts 1, 2, and 3) and the Guide for the Care and Use of Laboratory Animals (National Research Council, 2011).

### Test articles

For determination of PK, formulations were provided by Lexaria Bioscience Corp. (Kelowna, BC). DehydraTECH™ delivery technology includes a patented process by which long-chain fatty acids, high in oleic acid, are associated with CBD through a dehydration procedure. This technology is theorized to improve uptake of CBD by bypassing (or reducing) first-pass liver metabolism, allowing for higher concentrations of CBD to enter the circulatory system. Formulations tested in the present study differed in the type and quantity of long- and medium-chain fatty acids, and incorporation of certain adjunct/carrier ingredients, as well as the processing conditions applied in their production; their approximate compositions are described in pending and issued DehydraTECH™ patents. Analytical stock solutions (1.00 mg/mL of the free drug, CBD) were prepared in dimethylsulfoxide (DMSO) using nominal concentrations.

For determination of efficacy, CBDtech (mean potency = 29.73 mg/g) and vehicle were provided by Lexaria Bioscience Corp. (Kelowna, BC) as powdered compounds, and were stored at 2 to 8˚C protected from light. Once diluted in sterile water, working solutions (5 mg/mL) were stored at 2 to 8˚C or on wet ice for ≤ 12 h. Epidiolex® (100 mg/mL CBD) was purchased from SunSource Pharmaceuticals (Malvern, PA) as a liquid solution, and was stored at ambient temperature and used within 12 weeks of opening.

### Animals

Male Sprague Dawley rats for the PK (286–315 g body weight) and efficacy (4–6 weeks of age; 94.5–250.6 g body weight) studies were acquired from Charles River Laboratories (Wilmington, MA) and were quarantined for a minimum of seven days following arrival at the testing facility.

### Determination of plasma PK, brain distribution, and excretion

Animals were randomized to receive CBD in MCT oil or one of four CBD formulations employing DehydraTECH™ technology (*n* = 10 per group). All formulations were administered at 25 mg/kg CBD by oral gavage (p.o.). Plasma (up to 1 or 2 h), brain tissue (8 and 24 h) and urine and feces (up to 24 h) samples were collected.

Brain and feces samples were each homogenized (Virsonic 100 ultrasonic homogenizer) with 50:50 acetonitrile:water or 20:80 MeOH:water, respectively, prior to extraction. Samples were stored frozen (−80 °C) until analysis. Plasma, urine, and brain tissue samples were manually extracted via acetonitrile precipitation in a 96-well plate. Urine samples were diluted a minimum of fivefold into blank rat plasma prior to extraction. Diluted urine samples were quantified against the rat plasma calibration curve. To compare values relative to the DehydraTECH and control compositions, brain tissue and feces homogenates were quantified against calibration curves prepared in their respective matched blank tissue homogenate. Concentrations of the analyte, CBD, were determined by liquid chromatography with tandem mass spectrometry (LC–MS/MS; PE Sciex API4000).

### Determination of CBDtech efficacy in rodent models of seizure

Only the leading formulation, CBDtech (New DehydraTECH™ 2.0, formulation 6), was carried forth in efficacy studies. Study designs are summarized in Table 1.

**Table 1:** Summary of study designs for the determination of CBDtech efficacy in male Sprague Dawley rats following MES

Group	Treatment	Target Dose	Dosing Route, Frequency	N	Endpoints
Study 1. Determination of the ED of CBDTech following MES					
1	Vehicle	–	PO, single	7	Clinical observations, MSS, presence/absence of HLE, survival
2	CBDtech	≤ 100 mg/kg		7	
3	Epidiolex	≤ 100 mg/kg		7	
Study 2. Determination of the TPE of CBDtech following MES					
1	Vehicle	–	PO, single	8	Clinical observations, MSS, presence/absence of HLE, survival
2	CBDtech	75 mg/kg		8	
3	Epidiolex	75 mg/kg		8	
Study 3. Determination of ED50 of CBDtech following MES					
1	CBDtech	50 mg/kg	PO, single	8	Clinical observations, MSS
2	CBDtech	75 mg/kg		8	
3	CBDtech	100 mg/kg		8	

*BID* twice daily, *HLE* hind limb extension, *MES* maximum electroshock seizure, *MSS* maximal seizure severity, *N* number of animals, *PO* oral gavage

#### Study 1: Effective Dose (ED) of CBDtech following MES

The MES test is a widely used preclinical method to evaluate the efficacy of anticonvulsant drugs, particularly for tonic–clonic seizures (Löscher & Schmidt 1988). In this test, an electrical stimulus is applied to induce a seizure. The main endpoint observed is hind limb extension (HLE), where the animal’s hind limbs extend rigidly, indicating the spread of seizure activity through the motor pathways of the brain. Drugs that prevent HLE in the MES test are considered effective at blocking seizure propagation, often by stabilizing sodium channels, making this test a valuable tool for screening potential treatments for epilepsy.

Rats were randomly assigned to receive vehicle, CBDtech, or Epidiolex®. Animals were pretreated (1.5 ± 0.5 h) with a single p.o. dose of the assigned test article prior to MES induction. Pretreatment time was based on published literature regarding the biological activity of Epidiolex® (Patra et al. 2019). Clinical signs, maximal seizure severity, and suppression of HLE were assessed immediately following the MES test. Dosing began at 50 mg/kg in one animal and increased in 25 mg/kg increments in subsequent animals up to 100 mg/kg until an effective dose was determined.

#### Study 2: determination of the Time of Peak Efficacy (TPE) of CBDtech following MES

Rats were randomly assigned to receive vehicle, CBDtech or Epidiolex®. Using the effective dose determined in Study 1, MES was conducted at five timepoints (25 ± 5, 30 ± 10, 60 ± 15, 120 ± 20, and 360 ± 30 min) following a single administration of test articles. MSS was assessed at each timepoint. Clinical signs, maximal seizure severity, and suppression of HLE were assessed immediately following the MES test.

#### Study 3: median effective dose (ED50) of CBDtech following MES

Rats were randomly assigned to receive CBDtech at low, medium, or high dose (50, 75, or 100 mg/kg, respectively). MES was conducted at the TPE defined in Study 2 until at least two points were established between 0 and 100% protection. The ED50, the dose required to produce the desired endpoint in 50% of animals, in each test was determined using Probit analysis. Probit analysis is a commonly used parametric procedure that relies on linear regression following transformation of toxicity data for characterizing binomial response variables (Pum 2019).

### MES Induction

MES was induced using corneal electrical stimulation. Anesthetic/electrolyte solution (0.5% tetracaine hydrochloride in 0.9% saline) was applied to the eyes approximately 3 ± 2 min prior to placement of the corneal electrodes under brief manual restraint (up to 10 min). The stimulus (150 mA, 60 Hz, 0.2 s) was applied using an electro-convulsive device (Ugo Basile, model 57,800), inducing maximal seizures in the hind limbs with tonic extension. This stimulus was ~ 5–10 times higher than the individual animal electrical seizure threshold, eliminating bias stemming from daily fluctuations in seizure threshold. Animals were released at the moment of stimulation to permit seizure observation. Seizure score was determined using Racine's scale, which assesses the severity of seizures from 0 (no changes in behavior) to 5 (tonic–clonic seizure with full extension of fore-and hind-limbs)(Racine 1972). Animals were provided LabGel and supplemental fluids if dehydration was suspected.

### Statistical analyses

Pharmacokinetic parameters were determined with Phoenix WinNonlin (version 8.0) software using a non-compartmental model. The maximum plasma concentration (C_max_) and the time to reach maximum plasma concentration (t_max_) were observed from the data. The area under the time-concentration curve (AUC) was calculated using the linear trapezoidal rule with calculation to the last quantifiable data point. Any samples below the limit of quantitation (BLOQ; 0.5 ng/mL) were treated as zero for the purpose of analyses and were not used in the calculation of averages. Group differences in plasma PK parameters, brain concentration, and urine and feces percent recovery were determined by one-way analysis of variance (ANOVA) followed by Dunnett's multiple comparisons test using GraphPad Prism for macOS (version 10.4.0). All efficacy data were analyzed using Provantis (version 10.5.0.2). The ED50 was determined using Reed-Muench method (Reed & Muench 1938).

## Results

### Determination of CBDtech plasma PK, brain distribution, and excretion

No abnormal clinical signs were observed following dosing.

**Fig. 1:**
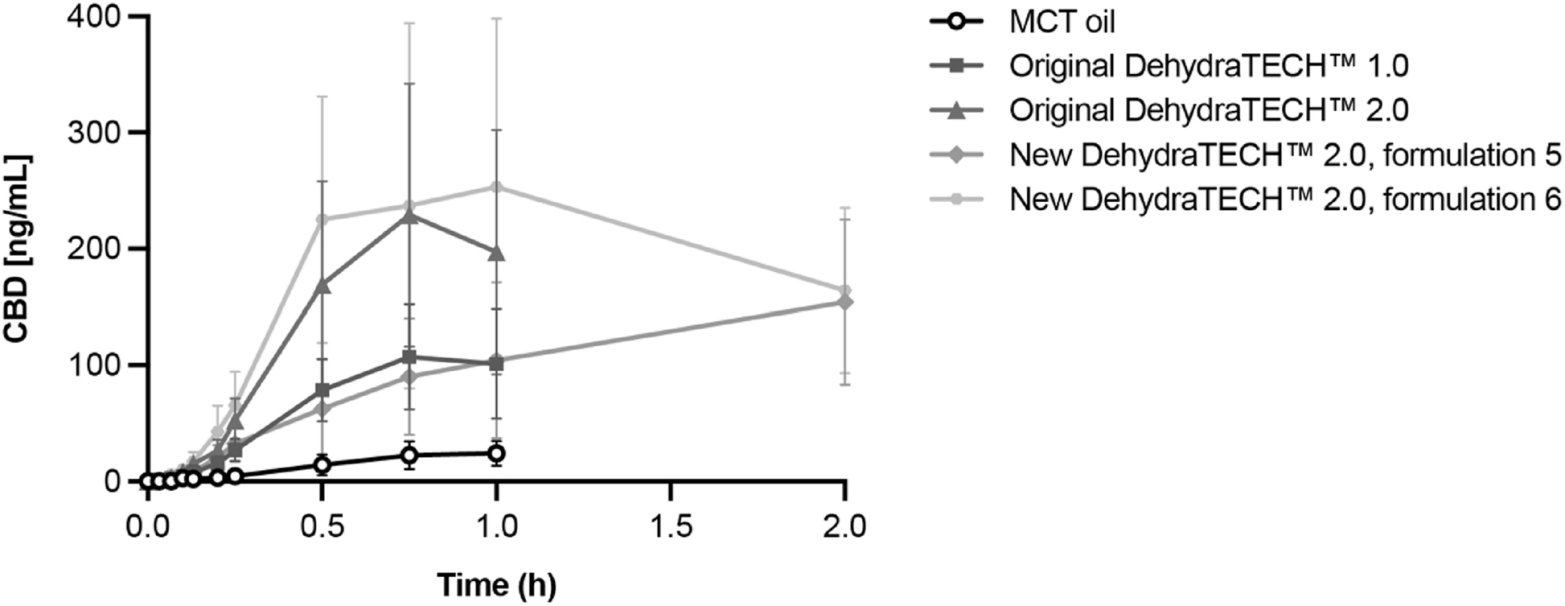
Mean ± SD plasma concentration of CBD after oral administration of various formulations of 25 mg/kg CBD in male Sprague–Dawley rats (*n* = 10 per group)

Plasma PK data are summarized in Table 2 and Fig. 1. With the exception of New DehydraTECH™ 2.0, formulation 5 (T_max_ = 1.68 h; *p* < 0.001), remaining formulations had a T_max_ that was less than one hour post dose and did not significantly differ from MCT control. C_max_ was significantly improved following administration of Original DehydraTECH™ 2.0 (807.6%; *p* < 0.001), New DehydraTECH™ 2.0, formulation 5 (515.2%; *p* = 0.007), and New DehydraTECH™ 2.0, formulation 6 (1064.5%; *p* < 0.001) as compared with the MCT control. Similarly, AUC_last_ was significantly improved following administration of Original DehydraTECH™ 2.0 (923.4%; *p* = 0.019), New DehydraTECH™ 2.0, formulation 5 (1322.8%; *p* < 0.001), and New DehydraTECH™ 2.0, formulation 6 (2707.3%; *p* < 0.001) as compared with the MCT control. Moreover, AUC_last_ was significantly improved following administration of New DehydraTECH™ 2.0, formulation 5 (190.0%; *p* = 0.032), and New DehydraTECH™ 2.0, formulation 6 (472.4%; *p* < 0.001) as compared with Original DehydraTECH™ 1.0. Finally, AUC_last_ was 174.5% improved following treatment with New DehydraTECH™ 2.0, formulation 6 as compared with Original DehydraTECH™ 2.0 (*p* < 0.001).

**Table 2:** CBD plasma pharmacokinetics after oral administration of various formulations of 25 mg/kg CBD in male Sprague–Dawley rats (*n* = 10 per group)

Test Article	t_max_ (hr)	C_max_ (ng/mL)	AUC_last_ (hr•ng/mL)	AUC_last_/dose (hr•kg•ng/mL/mg)
MCT Control	0.95 ± 0.11	25.8 ± 12.0	13.2 ± 6.8^a^	0.53 ± 0.27^a^
Original DehydraTECH™ 1.0	0.83 ± 0.21	112.2 ± 46.6	64.6 ± 23.7^a^	2.58 ± 0.95^a^
Original DehydraTECH™ 2.0	0.75 ± 0.12	234.6 ± 111.0*	134.7 ± 63.7*^a^	5.39 ± 2.55*^a^
New DehydraTECH™ 2.0, formulation 5	1.68 ± 0.53*	159.0 ± 68.3*	187.4 ± 94.6*^b^	7.5 ± 3.8*^b^
New DehydraTECH™ 2.0, formulation 6	0.88 ± 0.46	301.0 ± 146.7*	369.8 ± 171.9*^b^	14.8 ± 6.9*^b^

Values are mean ± SD. LLOQ = 0.5 ng/mL

^*^p < 0.05 as compared with MCT control; ^a^1 hour; ^b^2 hours

*AUC*_*last*_ area under the curve to the last observable time point, *CBD* cannabidiol, *C*_*max*_ maximum plasma concentration, *h* hours, *LLOQ* lower limit of quantification, *MCT* medium chain triglyceride, *ND* not determined, *SD* standard deviation, *t*_*max*_ time of maximum plasma concentration

Data from determination of CBD concentration in brain, urine, and feces are summarized in Table 3.

**Table 3:** CBD concentration in brain, urine, and fecal samples after oral administration of various formulations of 25 mg/kg CBD in male Sprague–Dawley rats (*n* = 5 per treatment group per timepoint)

Test Article	Matrix and Collection Time					
	Brain		Urine		Feces	
	Concentration (ng/g)		Recovery (% of dose)		Recovery (% of dose)	
	8 h	24 h	0-8 h	0-24 h	0-8 h	0-24 h
MCT Control	13.6 ± 9.1	ND	0.0019 ± 0.0020	0.0022 ± 0.0013	1.66 ± 1.68	3.38 ± 1.42
Original DehydraTECH™ 1.0	46.8 ± 12.3	2.5 ± 0.8	0.0002 ± 0.0001^c^	0.0009 ± 0.0007^a^	0.24 ± 0.21	13.46 + 6.79*
Original DehydraTECH™ 2.0	275.0 ± 154.9*	6.2 ± 2.0	0.0003 ± 0.0004^b^	0.0012 ± 0.0011	0.51 ± 0.88^b^	13.75 ± 3.87*
New DehydraTECH™ 2.0, formulation 5	49.7 ± 24.6	1.6^d^	0.0006 ± 0.0008^b^	0.0006 ± 0.0004^b^	0.005 ± 0.004^b^	5.38 ± 2.59
New DehydraTECH™ 2.0, formulation 6	120 ± 54	3.0 ± 1.2^c^	0.0005 ± 0.0003	0.0012 ± 0.0006	0.075 ± 0.088^a^	4.97 ± 1.77

Values are mean ± SD. LLOQ = 0.5 ng/mL. Samples where CBD was not detected were excluded from analyses

^*^p < 0.05 as compared with MCT control

^a^*n* = 4; ^b^*n* = 3; ^c^*n* = 2; ^d^*n* = 1

*CBD* cannabidiol, *h* hours, *LLOQ* lower limit of quantification, *MCT* medium chain triglyceride, *ND* not determined, *SD* standard deviation

Brain distribution was consistently higher following treatment with all DehydraTECH™ formulations, but was only significantly higher at the 8-h timepoint following administration of Original DehydraTECH™ 2.0 (1927.7%; *p* < 0.001). CBD was detected in urine and feces samples following all treatments. In urine, percent (%) recovery of CBD did not differ across treatments at either the 8- or 24-h timepoint. In feces, % recovery of CBD was significantly higher at the 24-h timepoint following administration of Original DehydraTECH™ 1.0 (298.7%; *p* = 0.002) and Original DehydraTECH™ 2.0 (307.3%; *p* = 0.001) as compared with the MCT control.

Given the above data, New DehydraTECH™ 2.0, formulation 6 (CBDtech) was selected as the lead formulation for subsequent efficacy work.

### Determination of CBDtech efficacy

**Fig. 2:**
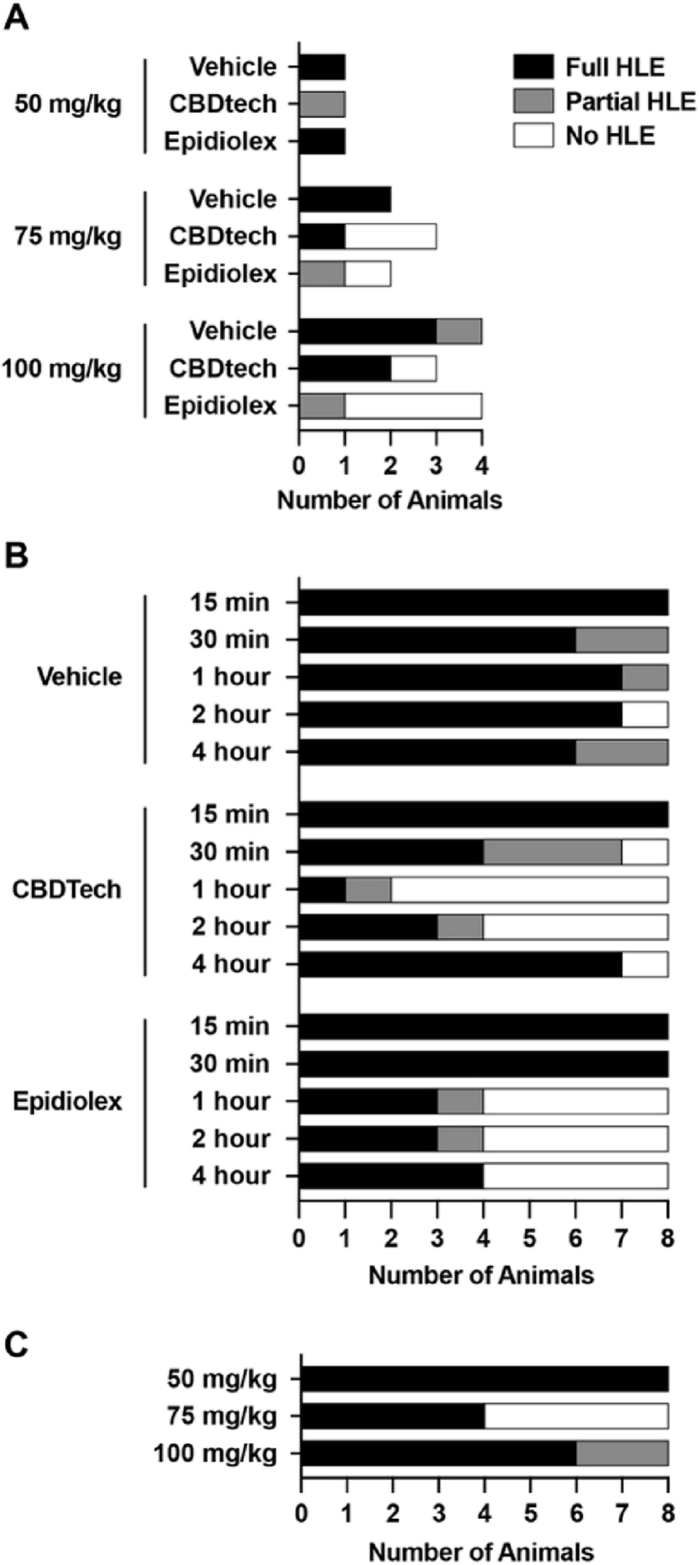
Determination of anticonvulsant activity of CBDtech in the maximal electroshock seizure model in male Sprague–Dawley rats. Number of animals per treatment/dose observed with full hind limb extension (HLE; black), partial HLE (grey), or no HLE (white) are shown for A) study 1, B) study 2, and 3) study 3

Anticonvulsant activity data from MES studies are summarized in Fig. 2.

#### Study 1: ED of CBDTech following MES

No abnormal clinical signs were observed following dosing; all animals were normal, bright, and alert across all dose levels for the duration of testing and survived all procedures. Following electroshock, the majority of animals presenting with partial or full HLE also presented with a seizure severity score ranging from 1–5 on the Racine scale. The most common observations were myoclonic jerks, repetitive head/neck movements with/without tail stiffening (stage 1), unilateral or incomplete clonic seizures (stage 2), and/or bilateral forelimb clonus with rearing (stage 3). Hyperactivity was observed in a select number of HLE presenting animals post MES test, characterized as a combination of running and/or bouncing activity.

MES testing was performed at a single timepoint (1.5 h) following test article administration. Recovery from the MES had an average duration of 30–40 s; animals without HLE recovered marginally quicker (10–40 s). Most animals made a full recovery within one minute post electroshock; however, one CBDtech-treated animal (100 mg/kg) presented with a sustained tonic seizure lasting approximately 12 min; the animal was upright on all four limbs and had normal respirations but was unresponsive to frequent stimulation.

Animals treated with 50 mg/kg Epidiolex® or vehicle presented with full HLE following MES test indicating no protection at this timepoint. Treatment with 50 mg/kg CBDtech in a single test subject resulted in partial HLE, characterized by partial or incomplete extension of one or both hind limbs. Treatment with 75 mg/kg of CBDtech revealed full protection (no HLE) in 66.6% of test subjects following MES test compared to 50% in the Epidiolex® group and 0% in the vehicle treated group. At the 100 mg/kg dose level, Epidiolex® exhibited the highest level of protection with 75% of animals presenting with no HLE compared to 33% in the CBDtech group and 0% in the vehicle treated group. Based on these results, the effective dose of CBDtech was determined to be 75 mg/kg.

#### Study 2: TPE of CBDtech following MES

No abnormal clinical signs were observed following dosing. All animals made a full recovery within one minute post electroshock.

MES testing was performed at five timepoints following test article administration at the effective dose (75 mg/kg) determined in Study 1. At the 15-min timepoint, all animals from all groups presented with full HLE. At the 30-min timepoint, animals treated with CBDtech exhibited absent (12.5%) or partial (37.5%) HLE; 0% of animals treated with Epidiolex® exhibited absent or partial HLE. At the one-hour timepoint, CBDtech yielded absent or partial HLE in 75 and 12.5% of animals, compared to 50 and 12.5% in the Epidiolex® group, respectively. At the two-hour timepoint, CBDtech and Epidiolex® had comparable efficacy, with 50 and 12.5% of animals showing full or partial protection, respectively. At the four-hour timepoint, Epidiolex® yielded absent HLE in 50% of animals, compared to 12.5% in the CBDtech group. Select animals in the vehicle treated group also presented with partial (12.5–25%) or full protection (12.5%) across timepoints, likely due to variability stemming from technical, biological (circadian or hormonally induced rhythms), and/or environmental factors. Based on these data, one hour was determined to be the TPE for CBDtech.

#### Study 3: ED50 of CBDtech following MES

No abnormal clinical signs were observed following dosing.

MES testing was performed at the TPE (60 min) following test article administration. Following 50 mg/kg CBDtech, HLE was observed across all animals. Following 75 mg/kg CBDtech, HLE was observed in 50% of animals, with the other 50% absent of HLE. Following 100 mg/kg CBDtech, HLE was observed in 75% of animals, with the remaining 25% displaying partial HLE. Seizure severity across all animals in Study 3 ranged from stage 1 to 3 on the Racine scale. The 75 and 100 mg/kg dosages were established as two points between 0 and 100% protection. The calculated ED50 was determined to be 75 mg/kg.

## Discussion

Despite the limited absorption and bioavailability of current oral CBD formulations, there is strong and growing interest in CBD’s potential therapeutic benefits. Here, we present data on the PK, brain distribution, excretion, and efficacy of novel CBD formulations utilizing DehydraTECH™ technology. This innovative approach is designed to bypass first-pass hepatic metabolism, enhancing systemic CBD exposure and maximizing the therapeutic potential of this botanically derived compound.

Our findings are consistent with previous human data wherein circulating CBD levels were significantly higher with 90 mg TurboCBD™, showing an 86% increase at 90 min and a 65% increase at 120 min post-exposure compared to a 90 mg CBD control formulation (Batinic et al. 2023; Patrician et al. 2019). Likewise, the DehydraTECH™ formulations in the present study demonstrated substantially improved systemic exposure, achieving ≥ 390% increased AUC_last_ compared to the MCT control. Others have previously reported improved PK with alternative oral CBD formulations. For example, administration of CBD (20 mg/kg) in a SEDDS formulation resulted in more rapid absorption and ~ 2.2 to 2.8-fold higher systemic exposure as compared with an MCT control in female Sprague–Dawley rats (Kok et al. 2022). In humans, dosing with a SEDDS CBD (25 mg) formulation led to 2.85-fold higher systemic exposure (AUC_0–8 h_) compared with an MCT control.

The anticonvulsant activity of CBD has previously been explored in a range of animal seizure and epilepsy models, including MES, following intraperitoneal (i.p.) administration. In one study, CBD was shown to block tonic extension seizures with an ED50 of 53.2 mg/kg in male rats, a dose well below the motor‐impairing dose (> 500 mg/kg) (Patra et al. 2019). Moreover, CBD (i.p.) was evaluated as part of the NIH’s Epilepsy Therapy Screening Program (ETSP), and demonstrated an ED50 of 88.9 mg/kg in the rat MES model (Klein et al. 2017). Similarly, in mice, the ED50 of CBD (i.p.) has been observed at 80–83.5 mg/kg (Gray et al. 2020; Klein et al. 2017; Patra et al. 2019; Socała et al. 2019; Wallace et al. 2001). These values are consistent with the ED50 of 75 mg/kg observed in the current study using an orally administered CBD formulation, underscoring the improved potency of CBDtech compared to traditional oral CBD preparations.

The MES test used in the present studies is an experimental model for generalized tonic–clonic seizures and, to a certain extent, partial convulsions. The model is highly reproducible, provides an indication of a compound’s ability to prevent seizure spread when all neuronal circuits in the brain are maximally active, and is considered to be electrophysiologically consistent with human seizures (White et al. 1995). The ED of CBDtech (75 mg/kg) in Study 1 resulted in marginally superior effects as compared to Epidiolex® at the same dose level; at the 100 mg/kg dose level Epidiolex® was twice as effective as CBDtech. At the established TPE (1 h), treatment with CBDtech resulted in full protection in 75% of animals, which was slightly greater than the 50% protection observed in the Epidiolex® group. Given the small number of animals per group for this study, full interpretation of these results is limited and warrants further study. Notably, despite no major discernable difference in T_max_ between DehydraTECH™ and MCT control, treatment with CBDtech resulted in faster effectiveness onset, as shown by partial (37.5% of animals) or complete (12.5% of animals) protection within 0.5 h post dose compared to no protection in animals treated with Epidiolex® at this timepoint. In contrast, Epidiolex® had sustained protection in 50% of the treated animals up to 4 h beginning 1-h post treatment.

Sex differences in the PK of CBD have been noted in several acute dosing studies (Arkell et al. 2021; Knaub et al. 2019, MacNair et al. 2023; Nadulski et al. 2005; Spindle et al. 2020). Only male animals were included in the present study; whether DehydraTECH™ CBD would demonstrate the same improvement in bioavailability and efficacy in female rodents remains to be elucidated. Moreover, the present study examined only immediate timepoints (up to 1–2 h post dosing) following acute dosing; future studies should explore later timepoints to better discern the elimination phase across treatments and repeated CBD exposures, which are more representative of human use patterns. Whereas the positive effects of DehydraTECH on CBD PK are unequivocal, further studies are needed to establish the mechanisms of action of this promising drug-delivery technology.

## Conclusions

In summary, these data demonstrate the improved bioavailability and anticonvulsant activity of CBD using advanced formulations employing DehydraTECH™. These findings support the further investigation of novel CBD formulations, to improve the efficacy of lipophilic drug candidates, including CBD.

## Data Availability

No datasets were generated or analysed during the current study.

## References

[CR1] Arkell TR, Kevin RC, Vinckenbosch F, Lintzeris N, Theunissen E, Ramaekers JG, McGregor IS. Sex differences in acute cannabis effects revisited: results from two randomized, controlled trials. Addict Biol. 2021:e13125. 10.1111/adb.13125. 10.1111/adb.13125 PMC9286641 34936167

[CR2] Atalay S, Jarocka-Karpowicz I, Skrzydlewska E. Antioxidative and anti-inflammatory properties of cannabidiol. Antioxidants (Basel, Switzerland). 2019;9(1):E21. 10.3390/antiox9010021. 10.3390/antiox9010021 PMC7023045 31881765

[CR3] Atsmon J, Heffetz D, Deutsch L, Deutsch F, Sacks H. Single-dose pharmacokinetics of oral cannabidiol following administration of PTL101: a new formulation based on gelatin matrix pellets technology. Clin Pharmacol Drug Dev. 2018;7(7):751–8. 10.1002/cpdd.408. 29125702 10.1002/cpdd.408

[CR4] Batinic A, Sutlović D, Kuret S, Matana A, Kumric M, Bozic J, Dujic Z. Trial of a novel oral cannabinoid formulation in patients with hypertension: a double-blind. Placebo-Controlled Pharmacogenetic Study Pharmaceuticals. 2023;16(5):645. 10.3390/ph16050645. 37242428 10.3390/ph16050645 PMC10223705

[CR5] Devinsky O, Cilio MR, Cross H, Fernandez-Ruiz J, French J, Hill C, Katz R, Di Marzo V, Jutras-Aswad D, Notcutt WG, Martinez-Orgado J, Robson PJ, Rohrback BG, Thiele E, Whalley B, Friedman D. Cannabidiol: Pharmacology and potential therapeutic role in epilepsy and other neuropsychiatric disorders. Epilepsia. 2014;55(6):791–802. 10.1111/epi.12631. 24854329 10.1111/epi.12631 PMC4707667

[CR6] Epidiolex Package Insert. 2018. https://pp.jazzpharma.com/pi/epidiolex.en.USPI.pdf.

[CR7] García-Gutiérrez MS, Navarrete F, Gasparyan A, Austrich-Olivares A, Sala F, Manzanares J. Cannabidiol: a potential new alternative for the treatment of anxiety, depression, and psychotic disorders. Biomolecules. 2020;10(11):E1575. 10.3390/biom10111575. 10.3390/biom10111575 PMC7699613 33228239

[CR8] Gray RA, Stott CG, Jones NA, Di Marzo V, Whalley BJ. Anticonvulsive properties of cannabidiol in a model of generalized seizure are transient receptor potential vanilloid 1 dependent. Cannabis Cannabinoid Res. 2020;5(2):145–9. 10.1089/can.2019.0028. 32656346 10.1089/can.2019.0028 PMC7347071

[CR9] Grotenhermen F, Russo E, Zuardi AW. Even high doses of oral cannabidol do not cause THC-like effects in humans: comment on Merrick et al. Cannabis and Cannabinoid Research 2016;1(1):102-112; 10.1089/can.2015.0004. Cannabis Cannabinoid Res. 2017;2(1):1–4. 10.1089/can.2016.0036. 10.1089/can.2016.0036 PMC5531368 28861499

[CR10] Klein BD, Jacobson CA, Metcalf CS, Smith MD, Wilcox KS, Hampson AJ, Kehne JH. Evaluation of cannabidiol in animal seizure models by the Epilepsy Therapy Screening Program (ETSP). Neurochem Res. 2017;42(7):1939–48. 10.1007/s11064-017-2287-8. 28478594 10.1007/s11064-017-2287-8

[CR11] Knaub K, Sartorius T, Dharsono T, Wacker R, Wilhelm M, Schön C. A Novel Self-Emulsifying Drug Delivery System (SEDDS) based on VESIsorb® formulation technology improving the oral bioavailability of cannabidiol in healthy subjects. Molecules (Basel, Switzerland). 2019;24(16):E2967. 10.3390/molecules24162967. 10.3390/molecules24162967 PMC6720748 31426272

[CR12] Kok LY, Bannigan P, Sanaee F, Evans JC, Dunne M, Regenold M, Ahmed L, Dubins D, Allen C. Development and pharmacokinetic evaluation of a self-nanoemulsifying drug delivery system for the oral delivery of cannabidiol. Eur J Pharm Sci. 2022;168: 106058. 10.1016/j.ejps.2021.106058. 34763088 10.1016/j.ejps.2021.106058

[CR13] Kumric M, Bozic J, Dujic G, Vrdoljak J, Dujic Z. Chronic effects of effective oral cannabidiol delivery on 24-h ambulatory blood pressure and vascular outcomes in treated and untreated hypertension (HYPER-H21-4): study protocol for a randomized, placebo-controlled, and crossover study. J Person Med. 2022;12(7):1037. 10.3390/jpm12071037. 10.3390/jpm12071037 PMC9322251 35887534

[CR14] Kumric M, Dujic G, Vrdoljak J, Svagusa K, Kurir TT, Supe-Domic D, Dujic Z, Bozic J. CBD supplementation reduces arterial blood pressure via modulation of the sympatho-chromaffin system: a substudy from the HYPER-H21–4 trial. Biomed Pharmacother = Biomed Pharmacother. 2023;160:114387. 10.1016/j.biopha.2023.114387. 10.1016/j.biopha.2023.114387 36780785

[CR15] Löscher W, Schmidt D. Which animal models should be used in the search for new antiepileptic drugs? A proposal based on experimental and clinical considerations. Epilepsy Res. 1988;2(3):145–81. 10.1016/0920-1211(88)90054-x. 3058469 10.1016/0920-1211(88)90054-x

[CR16] MacNair L, Kulpa J, Hill ML, Eglit GML, Mosesova I, Bonn-Miller MO, Peters EN. Sex differences in the pharmacokinetics of cannabidiol and metabolites following oral administration of a cannabidiol-dominant cannabis oil in healthy adults. Cannabis Cannabinoid Res. 2023. 10.1089/can.2022.0345. 10.1089/can.2022.0345 37267269

[CR17] Millar SA, Stone NL, Yates AS, O’Sullivan SE. A systematic review on the pharmacokinetics of cannabidiol in humans. Front Pharmacol. 2018;9. 10.3389/fphar.2018.01365. 10.3389/fphar.2018.01365 PMC6275223 30534073

[CR18] Nadulski T, Pragst F, Weinberg G, Roser P, Schnelle M, Fronk EM, Stadelmann AM. Randomized, double-blind, placebo-controlled study about the effects of cannabidiol (CBD) on the pharmacokinetics of Delta9-tetrahydrocannabinol (THC) after oral application of THC verses standardized cannabis extract. Ther Drug Monit. 2005;27(6):799–810. 16306858 10.1097/01.ftd.0000177223.19294.5c

[CR19] National Research Council. Guide for the care and use of laboratory animals (8th ed.). National Academies Press (US). 2011. http://www.ncbi.nlm.nih.gov/books/NBK54050/. 21595115

[CR20] Patra PH, Barker-Haliski M, White HS, Whalley BJ, Glyn S, Sandhu H, Jones N, Bazelot M, Williams CM, McNeish AJ. Cannabidiol reduces seizures and associated behavioral comorbidities in a range of animal seizure and epilepsy models. Epilepsia. 2019;60(2):303–14. 10.1111/epi.14629. 30588604 10.1111/epi.14629 PMC6378611

[CR21] Patrician A, Versic-Bratincevic M, Mijacika T, Banic I, Marendic M, Sutlović D, Dujić Ž, Ainslie PN. Examination of a new delivery approach for oral cannabidiol in healthy subjects: a randomized, double-blinded, placebo-controlled pharmacokinetics study. Adv Ther. 2019;36(11):3196–210. 10.1007/s12325-019-01074-6. 31512143 10.1007/s12325-019-01074-6

[CR22] Pum J. Chapter six—a practical guide to validation and verification of analytical methods in the clinical laboratory. In G. S. Makowski (Ed.). Adv Clin Chem. 2019;90:215–281. 10.1016/bs.acc.2019.01.006. Elsevier. 10.1016/bs.acc.2019.01.006 31122610

[CR23] Racine RJ. Modification of seizure activity by electrical stimulation. II. Motor seizure. Electroencephalogr Clin Neurophysiol. 1972;32(3):281–294. 10.1016/0013-4694(72)90177-0. 10.1016/0013-4694(72)90177-0 4110397

[CR24] Reed LJ, Muench H. A simple method of estimating fifty per cent endpoints. Am J Epidemiol. 1938;27(3):493–7. 10.1093/oxfordjournals.aje.a118408.

[CR25] Rubin R. The path to the first FDA-approved cannabis-derived treatment and what comes next. JAMA. 2018;320(12):1227–9. 10.1001/jama.2018.11914. 30193358 10.1001/jama.2018.11914

[CR26] Socała K, Wyska E, Szafarz M, Nieoczym D, Wlaź P. Acute effect of cannabidiol on the activity of various novel antiepileptic drugs in the maximal electroshock- and 6 Hz-induced seizures in mice: Pharmacodynamic and pharmacokinetic studies. Neuropharmacology. 2019;158: 107733. 10.1016/j.neuropharm.2019.107733. 31377197 10.1016/j.neuropharm.2019.107733

[CR27] Spindle TR, Cone EJ, Goffi E, Weerts EM, Mitchell JM, Winecker RE, Bigelow GE, Flegel RR, Vandrey R. Pharmacodynamic effects of vaporized and oral cannabidiol (CBD) and vaporized CBD-dominant cannabis in infrequent cannabis users. Drug Alcohol Depend. 2020;211: 107937. 10.1016/j.drugalcdep.2020.107937. 32247649 10.1016/j.drugalcdep.2020.107937 PMC7414803

[CR28] Wallace MJ, Wiley JL, Martin BR, DeLorenzo RJ. Assessment of the role of CB1 receptors in cannabinoid anticonvulsant effects. Eur J Pharmacol. 2001;428(1):51–7. 10.1016/s0014-2999(01)01243-2. 11779037 10.1016/s0014-2999(01)01243-2

[CR29] White HS, Johnson M, Wolf HH, Kupferberg HJ. The early identification of anticonvulsant activity: role of the maximal electroshock and subcutaneous pentylenetetrazol seizure models. Ital J Neurol Sci. 1995;16(1–2):73–7. 10.1007/BF02229077. 7642355 10.1007/BF02229077

[CR30] Wright M, Di Ciano P, Brands B. Use of cannabidiol for the treatment of anxiety: a short synthesis of pre-clinical and clinical evidence. Cannabis Cannabinoid Res. 2020;5(3):191–6. 10.1089/can.2019.0052. 32923656 10.1089/can.2019.0052 PMC7480724

[CR31] Zgair A, Wong JC, Lee JB, Mistry J, Sivak O, Wasan KM, Hennig IM, Barrett DA, Constantinescu CS, Fischer PM, Gershkovich P. Dietary fats and pharmaceutical lipid excipients increase systemic exposure to orally administered cannabis and cannabis-based medicines. Am J Transl Res. 2016;8(8):3448–59. 27648135 PMC5009397

